# Distinguishable Prognostic Signatures and Tumor Immunogenicity Between Pancreatic Head Cancer and Pancreatic Body/Tail Cancer

**DOI:** 10.3389/fonc.2022.890715

**Published:** 2022-06-08

**Authors:** Weiyu Ge, Jingyu Ma, Tiebo Mao, Haiyan Xu, Xiaofei Zhang, Shumin Li, Yongchao Wang, Jiayu Yao, Ming Yue, Feng Jiao, Yu Wang, Meng Zhuo, Ting Han, Jiong Hu, Xiao Zhang, Jiujie Cui, Liwei Wang

**Affiliations:** State Key Laboratory of Oncogenes and Related Genes, Shanghai Cancer Institute, Department of Oncology, Renji Hospital, School of Medicine, Shanghai Jiao Tong University, Shanghai, China

**Keywords:** pancreatic adenocarcinoma, pancreatic head cancer, pancreatic body/tail cancers, immunologic and hallmark gene sets, prognosis

## Abstract

**Background:**

Pancreatic head cancer and pancreatic body/tail cancer are considered to have different clinical presentations and to have altered outcomes.

**Methods:**

Ninety cases of pancreatic adenocarcinoma (PDAC) from our institution were used as a discovery set and 166 cases of PDAC from the TCGA cohort were used as a validation set. According to the anatomical location, the cases of PDAC were divided into the pancreatic head cancer group and the pancreatic body/tail cancer group. Firstly, the different biological functions of the two groups were assessed by ssGSEA. Meanwhile, ESTIMATE and CIBERSORT were conducted to estimate immune infiltration. Then, a novel anatomical site-related risk score (SRS) model was constructed by LASSO and Cox regression. Survival and time-dependent ROC analysis was used to prove the predictive ability of our model in two cohorts. Subsequently, an integrated survival decision tree and a scoring nomogram were constructed to improve prognostic stratification and predictive accuracy for individual patients. In addition, gseaGO and gseaKEGG pathway analyses were performed on genes in the key module by the R package.

**Results:**

Overall survival and the objective response rate (ORR) of patients with pancreatic body/tail cancer were markedly superior to those with pancreatic head cancer. In addition, distinct immune characteristics and gene patterns were observed between the two groups. Then, we screened 5 biomarkers related to the prognosis of pancreatic cancer and constructed a more powerful novel SRS model to predict prognosis.

**Conclusions:**

Our research shed some light on the revelation of gene patterns, immune and mutational landscape characterizations, and their relationships in different PDAC locations.

## Introduction

Pancreatic adenocarcinoma (PDAC) is a highly malignant tumor with a rapidly increasing incidence in recent years. According to estimates from the World Health Organization (WHO) in 2020 (1), PDAC accounts for 495,773 new cases and 466,003 cancer deaths, and is the seventh leading cause of cancer death in both men and women worldwide. In addition, the mortality to incidence of PDAC is about 0.94, ranking first among all common tumors (2). The 5-year survival rate is 10% at present, and is projected to become the second leading cause of cancer death in USA by 2030 (3).

Accounting for the poor outcome of PDAC, numerous investigations have been done to detect and evaluate the prognostic factors of PDAC, so that follow-up care and management can be planned appropriately. Tumor size and histological characteristics are well-known as major prognostic factors for PDAC (4, 5). Moreover, gender, age, pathological stage, cancer antigen 19-9 (CA19-9) level, and genomic analysis have been proposed as significant prognostic factors for PDAC in recent studies (6–8). The importance of tumor location of PDAC to prognosis has been demonstrated as well (9–12).

Different locations of tumor usually contribute to different clinical features and prognoses, so that different therapeutic methods are required depending on the tumor locations. Approximately 60%–70% of pancreatic tumors occur in pancreatic head or neck, which tend to cause biliary obstruction, giving rise to the classic appearance of painless jaundice. Tumors of the pancreatic body are more likely to invade local vascular structures including the portal vein, celiac, hepatic, and superior mesenteric vessels, leading to back pain on presentation (3). Pancreatic tail tumors can often grow latently because of fewer anatomical neighbors and are prone to be advanced at the time of diagnosis with symptoms from sites of metastases (13). When it comes to resectable and borderline resectable PDAC, pancreatic head tumors are typically treated with a pancreaticoduodenectomy, while tumors in the body or the tail of the pancreas can be resected with a distal pancreatectomy, often combined with splenectomy (14, 15). Based on the principle of individualized treatment, distinguishing systemic therapies related to the location of PDAC are needed.

Several studies have indicated that the anatomical site of PDAC may have a different biological nature and survival, while the outcome is controversial (9–12). Some studies supported that pancreatic head cancer has a better overall patient survival than pancreatic body/tail tumor, due to the earlier diagnosis of pancreatic head cancer and the high resection rate (9, 11, 16). However, some studies showed that the prognosis of pancreatic body/tail cancer is better than that of pancreatic head cancer (17, 18). The reasons of the divided opinions and the mechanism of how location affect prognosis remain to be explored.

Emerging studies have identified that molecular subtypes of PDAC based on genomic analyses is associated with prognosis and implication of therapies (19). The differentially expressed genes (DEGs) and mutation signatures of pancreatic head tumors and pancreatic body/tail tumors have been analyzed as well (20). Yet, the exact genetic differences between pancreatic head cancer and pancreatic body/tail cancer have not been fully elucidated, and there was no anatomical site-based prognostic model available that can be used to identify high-risk patients in PDAC.

Immune checkpoint inhibitors have offered promise in the treatment of a wide range of malignancies, but unfortunately, this clinical benefit has not yet translated to pancreatic ductal adenocarcinoma (21), due to the complexity of this tumor and the highly immunosuppressive microenvironment (22). In addition, the efficacy of immunotherapy is closely related to the components of the tumor microenvironment (TME) (23). We would like to investigate whether there are any differences in the infiltration level of immune cells between pancreatic head cancer and pancreatic body/tail cancer, which may be helpful for choosing the proper immunotherapy approach as well.

Therefore, the main purpose of this study is to analyze the different immune microenvironment, and the various hallmarks and mutation signatures of pancreatic head cancer and pancreatic body/tail cancer, and to find out the significant mechanisms that are related to PDAC sites. In addition, we established and validated a robust anatomical site-related risk score (SRS) model to predict prognosis for PDAC patients. Finally, an integrated model based on the gene signature and clinicopathological features was developed to improve the predictive power and accuracy.

## Materials and Methods

### Data Source and Patient Selection

We obtained information on 90 primary curative resection PDAC patients at Shanghai Renji Hospital (Shanghai, Shanghai Jiao Tong University School of Medicine, China) as a discovery set. The criteria for screening these patients are the first diagnosis and first treatment in the Department of Oncology of our hospital between 2017 and 2021, and the patients with pancreatic cancer who can track the complete clinical information. This standard reduces the deviation of the results due to the difference in diagnosis and treatment.

According to the anatomical location, the patients were divided into 41 cases of pancreatic head cancer and 49 cases of pancreatic body/tail cancer, including clinical features, outcomes of chemotherapy [nab-paclitaxel (a novel albumin-bound, solvent-free, and water-soluble formulation of paclitaxel) combined with gemcitabine], and detection indicators (before first-line treatment) of PDAC patients in the peripheral blood samples (**Table 1**).

**Table 1:** Clinical features and detection indicators of PDAC patients in the peripheral blood samples.

Features	*n* (%)
**Type, *n* (%)**	
Head	49 (54.4%)
Tail	41 (45.6%)
**Gender, *n* (%)**	
Female	34 (37.8%)
Male	56 (62.2%)
**Age, *n* (%)**	
<65	52 (57.8%)
≥65	38 (42.2%)
**TNM Stage, *n* (%)**	
I–II	22 (25%)
III–IV	66 (75%)
**CEA, *n* (%)**	
<5 ng/ml	39 (47.6%)
≥5 ng/ml	43 (52.4%)
**CA199, *n* (%)**	
<100 U/ml	14 (17.1%)
≥100 U/ml	68 (82.9%)
**CA125, *n* (%)**	
<35 U/ml	36 (44.4%)
≥35 U/ml	45 (55.6%)
**CA724, *n* (%)**	
<7 U/ml	47 (62.7%)
≥7 U/ml	28 (37.3%)
**CA50, *n* (%)**	
<24 U/ml	9 (12%)
≥24 U/ml	66 (88%)
**Total bile acid, *n* (%)**	
<10 μmol/L	69 (81.2%)
≥10 μmol/L	16 (18.8%)
**Cholesterol, *n* (%)**	
<5.68 mmol/L	61 (88.4%)
≥5.68 mmol/L	8 (11.6%)
**Total bilirubin, *n* (%)**	
<17.1 μmol/L	62 (72.9%)
≥17.1 μmol/L	23 (27.1%)
**Direct bilirubin, *n* (%)**	
<6 μmol/L	62 (72.9%)
≥6 μmol/L	23 (27.1%)
**ALT, *n* (%)**	
<40 IU/ml	63 (74.1%)
≥40 IU/ml	22 (25.9%)
**AST, *n* (%)**	
<45 IU/ml	68 (80%)
≥45 IU/ml	17 (20%)
**GGT, *n* (%)**	
<50 IU/ml	37 (43.5%)
≥50 IU/ml	48 (56.5%)
**ALT, *n* (%)**	
<160 IU/ml	63 (74.1%)
≥160 IU/ml	22 (25.9%)

PDAC, pancreatic adenocarcinoma.

### Validation and Training Cohorts

RNA-seq expression profiles, clinical data, and mutation data were downloaded and collected from TCGA (https://xenabrowser.net/datapages/); RNA-seq expression profiles contain 105 pancreatic head cancer cases and 19 pancreatic body/tail cancer cases. Clinical information contains 136 pancreatic head cancer cases and 30 pancreatic body/tail cancer cases, including age, gender, overall survival (OS), disease-free survival (DFS), and TNM stage. In order to verify the results in different cohorts, 124 TCGA-PDAC patients were divided into validation (*n* = 62) and training cohorts (*n* = 62) with “survival”, “caret”, “glmnet”, and “survminer” R packages. To ensure that these patients of pancreatic head cancer and pancreatic body/tail cancer were comparable, the Kaplan–Meier method was used to draw survival curves, and the log-rank test was performed to evaluate survival difference. The criterion for statistical significance was *p* < 0.05 on the above analysis (**Figure 2A**).

### Prognostic Factors of Pancreatic Head Cancer and Pancreatic Body/Tail Cancer and Construction of Cox Regression Models

To explore the different prognostic molecules of pancreatic head cancer and pancreatic body/tail cancer, the impact of the different molecular expression levels on the prognosis of patients with pancreatic head cancer and pancreatic body/tail cancer was investigated by univariate Cox regression analysis. The molecules that affect the survival of the patients with pancreatic head cancer and pancreatic body/tail cancer were preliminarily screened. Multiple stepwise Cox regression analysis was performed for potentially relevant genes in the preliminary screening, and the influence of these molecules on the survival time and the survival outcome was also analyzed (**Table 2**). *p* < 0.05 served as a standard condition for the above screening.

**Table 2:** Association of overall survival with clinicopathological factors and tumor anatomical location of pancreatic cancer patients in TCGA-independent validation cohorts.

Characteristics			Univariate analysis				Multivariate analysis		
		HR	HR.95L	HR.95H	*p*-value	HR	HR.95L	HR.95H	*p*-value
**Primary tumor site**		0.393	0.201	0.769	0.0064	1.383	1.249	1.531	<0.0001
Head vs.		136 (166)				136 (166)			
Tail/Body		30 (166)				30 (166)			
**Gender**		0.736	0.48396	1.119	0.152	0.732	0.452	1.187	0.206
Female vs.		74 (166)				74 (166)			
Male		92 (166)				92 (166)			
**Age**		0.695	0.451	1.070	0.098	1.023	0.998	1.049	0.072
<65 vs.		74 (166)				74 (166)			
≥65		92 (166)				92 (166)			
**TNM Stage**		0.864	0.349	2.144	0.753	0.888	0.518	1.521	0.665
I–II vs.		153 (166)				153 (166)			
III–IV		13 (166)				13 (166)			

The testing of the proportional hazard assumption: log(−log) plot.

### ssGSEA Analysis of Pancreatic Head Cancer and Pancreatic Body/Tail Cancer

To reveal the variances in biological functions between pancreatic head cancer and pancreatic body/tail cancer, the single-sample gene set enrichment analysis (ssGSEA) algorithm based on the transcriptome data and corresponding gene sets retrieved from the Molecular Signatures Database (MSigDB) (24, 25) was applied. As previously described, the Molecular Function (MF) analysis and Reactome pathway analysis were performed by the R package “clusterProfiler” (26, 27). All genes with different expression levels were divided into a pancreatic head cancer and a pancreatic body/tail cancer group for comparison. The gene set “h.all.v7.2.symbols.gmt” was chosen as the reference gene set; adjusted *p* < 0.01 was regarded as statistical significance.

### Immune Infiltration Analysis of Pancreatic Head Cancer and Pancreatic Body/Tail Cancer

The two algorithms named ESTIMATE (28), and CIBERSORT (29) were used to quantify the relative or absolute abundance of immune and stromal cell populations between pancreatic head cancer and pancreatic body/tail cancer with transcriptome data. The tumor purity of PDAC samples was estimated using the R package “ESTIMATE”. The immune infiltration was defined as the sum of absolute abundance of CD8+T cells, CD4+T cells, regulatory T cells (Tregs), macrophages, myeloid-derived suppressor cells (MDSCs), and DC cells. The cytolytic activity (CYT) score was defined as the geometric mean of PRF1 and GZMA (30).

### Establishment of the Anatomical Site-Related Risk Score

DEGs of pancreatic head cancer and pancreatic body/tail cancer were identified using the R package “limma” for microarray data or “DESeq2” for RNA-seq read count data. DEGs were defined with the thresholds of adjusted *p* < 0.05 and |log2 Fold Change| > 1.0 (31). Then, using the R package “Cox-PH”, the values of hazard ratio (HR) and *p* were calculated for each significant differential gene, and candidate genes with a *p*-value < 0.05 were used as the input of the least absolute shrinkage and selection operator (LASSO) Cox regression model. LASSO regularization adds a penalty parameter (λ) to the Cox regression model, and this action can lead to zero coefficients; i.e., some of the candidate genes were completely neglected for the evaluation of output (32). In our analysis, 5 genes retained their Cox coefficients after LASSO regularization (**Figures 3A, B**). Based on their expression values and Cox coefficients, the immune-related risk score (SRS) of each sample was calculated as follows:


$$\text{SRS}=\sum_{i}\text{Coefficient }\left(m\text{RNAi}\right)\;\times \text{ Expression}\left(m\text{RNAi}\right)$$

The screened molecules were used to construct the prognostic index (PI) model, and univariate analysis was done to determine the prognostic power in the SRS-high group and the SRS-low group (**Figures 4A, B**). The “survcutpoint” function of the R package “survminer” was used to determine the optimal cutoff point based on the maximal log-rank statistics. Multivariate Cox regression analysis was performed to evaluate the risk significance of each variable for survival. Time-dependent receiver operating characteristic (tROC) analysis was performed to measure the predictive power with the R package “survival ROC” (33), and the areas under the curve at different time points [AUC(t)] of all the variables were compared. Meta-analysis (*I* ^2^ > 30%, random-effects model) was performed to evaluate the prognostic value in the pooled cohort. Based on SRS and clinicopathological features, recursive partitioning analysis was performed to build an integrated survival decision tree for risk stratification with the R package “rpart”. Using the R package “rms”, a scoring nomogram was generated with detailed parameters including gender, KARS and TP53 histological variant, age, TNM stage, and SRS.

### Enrichment Analysis of SRS

To reveal the correlation between the different biological functions and SRS, the gene set enrichment analysis (GSEA) method was used for the analysis in this study (34). Gene Ontology (GO) enrichment and Kyoto Encyclopedia of Genes and Genomes (KEGG) pathway analysis were performed on genes in the key module by the R package “clusterprofiler” (35). After setting the criteria of adjusted *p* < 0.01, GO terms and KEGG pathways were visualized by the package “ggplot2”.

### Nomogram Construction

We performed univariate analysis on clinicopathological parameters and our signature in the PDAC-TCGA cohort. The significant prognostic variables (*p* < 0.05) were subsequently incorporated into multivariate Cox regression analysis. The R package “rms” was utilized to build the nomogram adopting variables with predictive significance in multivariate analysis (*p* < 0.05). Calibration curves were used to assess the consistency between predicted and actual survival outcome. Furthermore, time-dependent ROC curves were applied to compare the predictive accuracy of nomogram, gene risk model, and clinicopathological factors.

### Additional Bioinformatic and Statistical Analyses

DEGs were identified using the R package “limma” for microarray data or “DESeq2” for RNA-seq read count data. Student’s *t*-test or one-way analysis of variance (ANOVA) was used to analyze differences between groups in variables with a normal distribution. Categorical variables between the two groups were compared using the chi-square test. A value of *p* < 0.05 was considered statistically significant. IBM SPSS Statistics 20 (IBM Corp., Armonk, NY, USA), GraphPad Prism 8.0 (GraphPad Software Inc, San Diego, CA, USA), Stata 12 (StataCorp LLC, TX, USA), and R software (version 3.6.2, http://www.r-project.org) were used to analyze data and plot graphs.

## Results

### Differential Clinicopathological Characteristics and Outcomes of Initial Therapy in Head and Body/Tail Pancreatic Cancers

**Figure 1:**
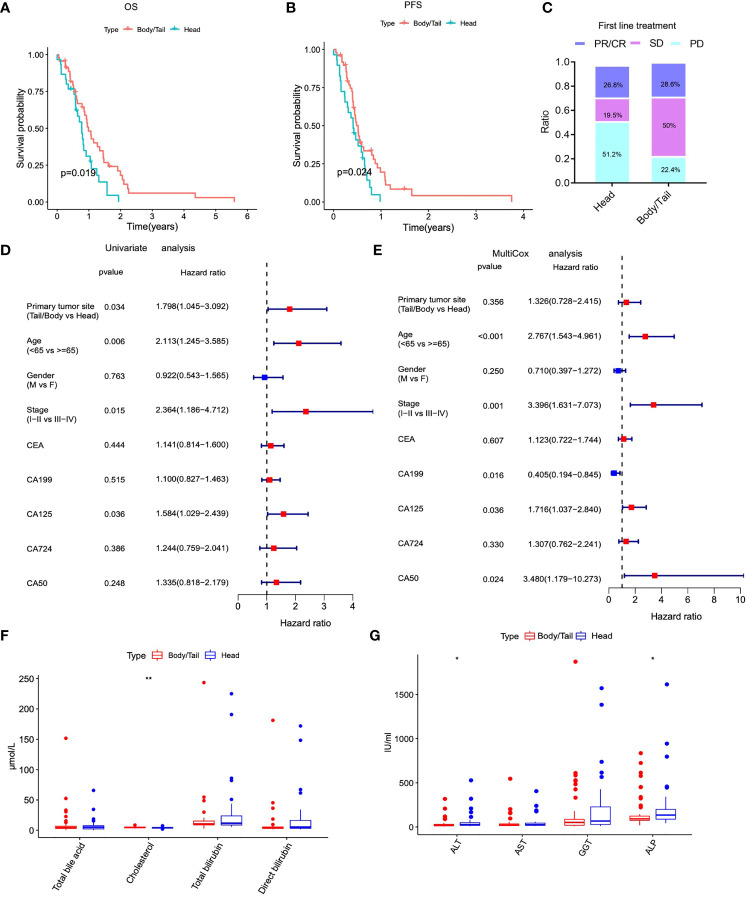
The anatomical location of PAAD was closely related to clinicopathological characteristics and outcomes of initial therapy. **(A)** Kaplan–Meier analysis showed that patients of pancreatic head cancers exhibited worse OS. **(B)** Kaplan-Meier (K-M) survival analysis showed that patients of pancreatic head cancer exhibited worse PFS. **(C)** The ORR of worse outcomes after first line treatment was greatly elevated in pancreatic head cancer group. **(D)** Univariate cox regression analysis demonstrated that primary tumor site was an independent risk factor for OS. **(E)** Multivariate cox regression analysis demonstrated that primary tumor site was an independent risk factor for OS. **(F–G)** The levels of important biochemical indexes of peripheral blood between pancreatic head cancer group and pancreatic body/tail cancer group in our institution’s data. PAAD, pancreatic adenocarcinoma; OS, overall survival; PFS, progression free survival; ORR: objective response rate; PD, progressive disease; PR, partial remission, SD, stable disease; HR, hazard ratio; **p < 0.01, *p < 0.05 and not significant (p < 0.05) by repeated measures with Wilcoxon test.

The information on PDAC patients from our institution included in the current study is displayed in **Table 1**. Firstly, the Kaplan–Meier survival analysis showed that the anatomical location of cancer was closely related to the prognosis of patients with PDAC. The OS (*p* = 0.019) and DFS (*p* = 0.024) of patients with pancreatic body/tail cancer was superior to those with pancreatic head cancer (**Figures 1A, B**). Subsequently, treatment information and clinical outcomes of patients from our institution were used to validate the result. After the initial first-line treatment, the objective response rate (ORR) of complete remission (CR) or partial remission (PR) in the pancreatic body/tail cancer group was greatly improved compared with the pancreatic head cancer group (**Figure 1C**). Moreover, we analyzed clinical variable information and tumor biomarkers of peripheral blood samples before first-line treatment from our institution, among tumor anatomical site, various clinicopathological variables (age, gender, and stage), and biomarkers (CA125, CEA, CA199, and CA724) on a log10 scale. Univariate and multivariate Cox regression modeling demonstrated that primary tumor site (uniCox: HR = 1.798, *p* = 0.034; multiCox: HR = 1.326, *p* = 0.356), age (uniCox: HR = 2.113, *p* = 0.006; multiCox: HR = 2.767, *p* < 0.001), stage (uniCox: HR = 2.364, *p* = 0.015; multiCox: HR = 3.396, *p* = 0.001), and CA125 (uniCox: HR = 1.584, *p* = 0.036; multiCox: HR = 1.716, *p* = 0.036) were independent risk factors for OS at the Renji Hospital discovery set. However, there was no significant difference in the Chi-square test of gender (uniCox: HR = 0.922, *p* = 0.763; multiCox: HR = 0.710, *p* = 0.250), CEA (uniCox: HR = 1.141, *p* = 0.444; multiCox: HR = 1.123, *p* = 0.607), CA199 (uniCox: HR = 1.100, *p* = 0.515; multiCox: HR = 0.405, *p* = 0.016), CA724 (uniCox: HR = 1.244, *p* = 0.386; multiCox: HR = 1.307, *p* = 0.330), and CA50 (uniCox: HR = 1.335, *p* = 0.248; multiCox: HR = 3.480, *p* = 0.024) of pancreatic head cancer and pancreatic body/tail cancer (**Figures 1D, E**). In addition, the levels of ALT (*p* = 0.0102) and ALP (*p* = 0.0374) in peripheral blood samples of the pancreatic head cancer group were significantly higher than those of the pancreatic body/tail cancer group, but cholesterol was significantly reduced (*p* = 0.0038) in the pancreatic head cancer group (**Figures 1F, G**).

**Figure 2:**
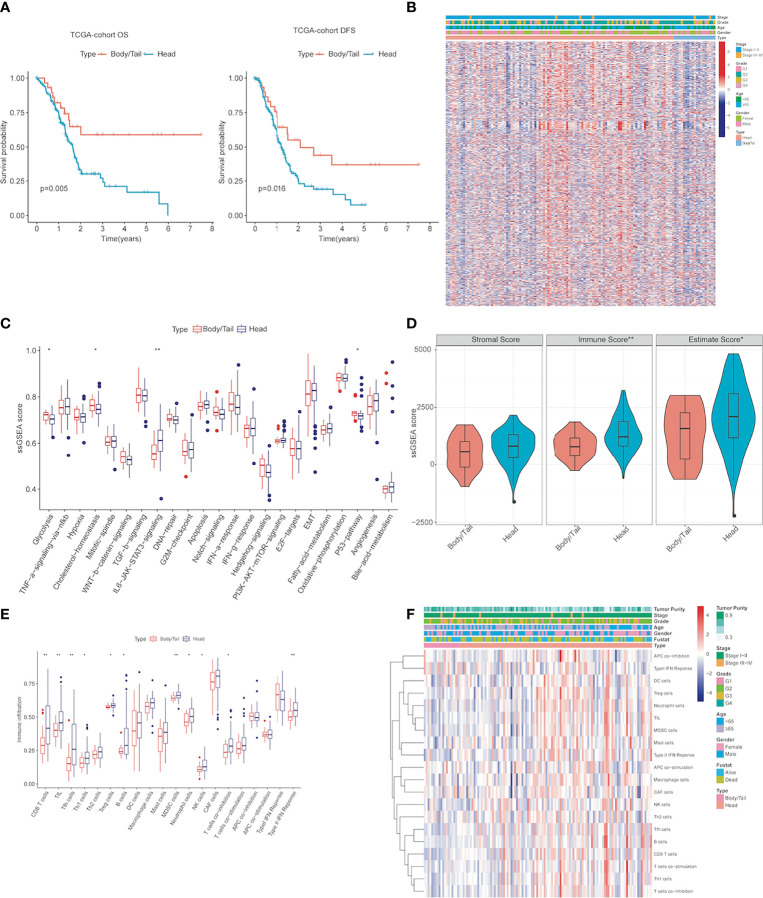
The activity changes of immune microenvironment and hallmark pathway in pancreatic head cancer and pancreatic body/tail cancer were identified **(A)** Kaplan–Meier survival analysis of the head and body/tail of pancreatic cancers patients’ OS and DFS in the TCGA validation cohorts. **(B)** Heatmaps of the upregulated and DEGs downregulated DEGs between pancreatic head cancer and pancreatic body/tail cancer in TCGA. **(C)** ssGSEA analysis demonstrated that various cancer hallmarks exhibited significantly higher activity in pancreatic head cancer group compared with pancreatic body/tail cancer group. **(D)** pancreatic head cancer was characterized with significantly higher immune score. **(E)** Most of the 20 immune cell types were differentially distributed between the two groups. **(F)** A comprehensive heatmap illustrated the differences of the immune infiltration of the tumor microenvironment between the two groups. ssGSEA, single-sample gene set enrichment analysis; **p < 0.01, *p < 0.05 and not significant (p < 0.05) by repeated measures with Wilcoxon test.

**Table 2** shows univariate analysis and multivariate analysis of 166 PDAC patients with clinicopathological factors in TCGA-independent validation cohorts. Furthermore, K-M prognostic analysis surely verified that pancreatic body/tail cancer has a better prognosis compared with pancreatic head cancer (OS *p* = 0.005, DFS *p* = 0.016, **Figure 2A**) using TCGA validation cohorts. These lines of evidence demonstrated that patients with pancreatic head cancer have a worse prognosis and ORR of the initial therapy.

### Distinct Immune Characteristics and Gene Patterns Were Observed Between Pancreatic Head Cancer and Pancreatic Body/Tail Cancer

Genetic patterns and immune characteristics are the two most important factors affecting the prognosis of PDAC patients (20, 23). Therefore, we analyzed the differences in expression profile and immune microenvironment between pancreatic head cancer and pancreatic body/tail cancer. There were 826 significant DEGs (|log2 Fold Change| > 0.5, *p* < 0.05), of which 603 genes were upregulated and 223 genes were downregulated (**Supplementary Table S1**). A comprehensive heatmap illustrates the different gene patterns (**Figure 2B**). Based on ssGSEA scores of cancer hallmarks in the TCGA-PDAC discovery cohort, the ssGSEA scores of each hallmark were calculated and ranked. The ssGSEA demonstrated that there were 4 significant hallmarks (FDR < 0.05), namely, glycolysis, cholesterol-homeostasis, IL6/JAK/STAT3 signaling, and P53 pathway (**Figure 2C**), which may be important mechanisms for the difference in prognosis between them.

Then, to evaluate the infiltrating levels of immune cells, tumor purity, and stromal cells involved in the TME, CIBERSORT and ESTIMATE were applied based on the transcriptome data of the 124 PDAC samples. **Figure 2D** illustrates that the immune score was notably higher in the pancreatic head cancer group than the body/tail pancreatic cancer group (*p* = 0.0074); however, stromal scores did not change noticeably. Subsequently, pancreatic head tumors demonstrated an immunosuppressive phenotype with more infiltrations of regulatory T cells (Tregs), myeloid-derived suppressor cells (MDSCs), tumor-associated macrophages (TAMs), and high expressions of genes negatively regulating anti-tumor immunity, but the infiltration of CD8T cells and NK cells also increased (**Figures 2E, F**). These lines of evidence indicated significant differences in gene patterns and tumor immune disorder between the two groups.

### Establishment and Validation of Site-Related Risk Score in PDAC Patients

**Figure 3:**
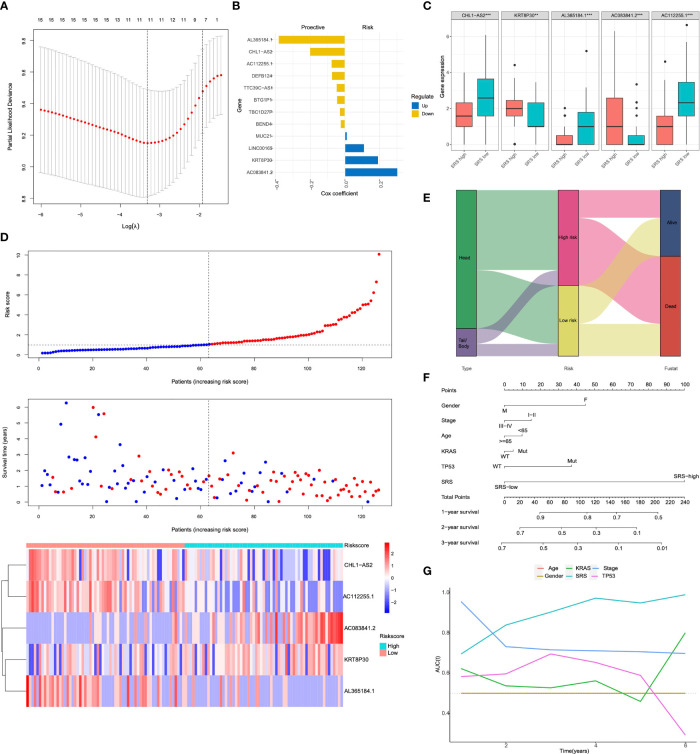
The anatomical site-related risk score (SRS) establishment **(A)** The LASSO Cox regression model was used to identify the most robust markers with an optimal λ value of 0.0617. **(B)** Distribution of LASSO coefficients of the anatomical site-related gene signature. **(C)** Expression levels of 5 selected genes between SRS-high and SRS-low tissues in TCGA database. **(D)** SRS model of the head and body/tail of pancreatic cancers. The top section showed the survival status of PAAD patients. Y-axis represented survival time of each patient. The middle section of figure showed the SRS. X-axis represented the patient IDs, which were ranking by SRS score from low to high. The heatmap of bottom showed CHL1-AS2, KRT8P30, AL365184.1, AC083841.2 and AC112255.1 expression in each PAAD patients. **(E)** a Sankey diagram depicted the flow from two different anatomical site to SRS and pathological fustate, in which the width of the flow rate is proportional to the patient number. **(F)** A nomogram was constructed to quantify risk assessment for individual patients. **(G)** tROC analysis demonstrated that the nomogram was the most stable and powerful predictor for OS among all the clinical variables. LASSO, least absolute shrinkage and selection operator; SRS, the anatomical site-related risk score; ROC, receiver operating characteristic; tROC, time-dependent receiver operating characteristic.

In order to screen the hub genes affecting the prognosis of pancreatic cancer, a univariate Cox proportional-hazards (Cox-PH) regression model was performed for the above DEGs in pancreatic head cancer and pancreatic body/tail cancer using the R package “survival”. In addition, the LASSO Cox regression model was used to identify the most robust markers for prognosis. With a threshold of *p*-value for univariate Cox <0.01, 12 promising candidates (8 protective and 4 risk markers) were identified (**Figure 3A**). An ensemble of 5 biomarkers (CHL1A-S2, KRT8P30, AL365184.1, AC083841.2, and AC112255.1) remained with their individual nonzero LASSO coefficients, and the distribution of LASSO coefficients of the gene signature is shown in **Figure 3B** and **Supplementary Table S2**. Finally, the SRS formula was established as follows: ∑_i_ Coefficient (mRNAi) × Expression (mRNAi). The expression level of each gene was log2 normalized. According to the median of risk score, patients were divided into SRS-high and SRS-low risk groups. The expression level of KRT8P30, AL365184.1, and AC083841.2 was significantly higher in the SRS-high risk group than in the SRS-low risk group (*p* < 0.001), while the expression level of CHL1-AS2 and AC112255.1 was reversely downregulated in the SRS-high risk group (*p* < 0.001) (**Figure 3C**). A prognostic index model for pancreatic head cancer and pancreatic body/tail cancer was constructed using these five genes; the expression data of these five genes were introduced into the PI equation (**Figure 3D**). Next, we proposed an integrated prognostic model *via* the combination of SRS and other clinicopathological features to improve risk stratification and to personalize risk assessment. A total of 124 TCGA-PDAC patients with full-scale clinical annotations consisting of gender, age, pathological stage, KRAS status, TP53 status, and SRS were extracted to construct integrated prognostic models, and we observed that most pancreatic head cancer samples were exclusively classified to high risk while half pancreatic body/tail cancer samples were classified to low risk (**Figure 3E**). With a goal of quantifying the risk assessment for individual PDAC patients, a personalized scoring nomogram was generated to predict 1- and 3-year OS probability with the six parameters (**Figure 3F**). Furthermore, time-dependent ROC analysis demonstrated that the SRS nomogram exhibited much more powerful capacity of survival prediction compared with other conventional clinicopathological features, with an average AUC above 0.8 during a follow-up of 6 years (**Figure 3G**). Generally, the integrated prognostic models could greatly optimize risk stratification and predict OS for PDAC patients accurately.

### SRS Serves as a Risk Factor for Overall Survival in Each Cohort

**Figure 4:**
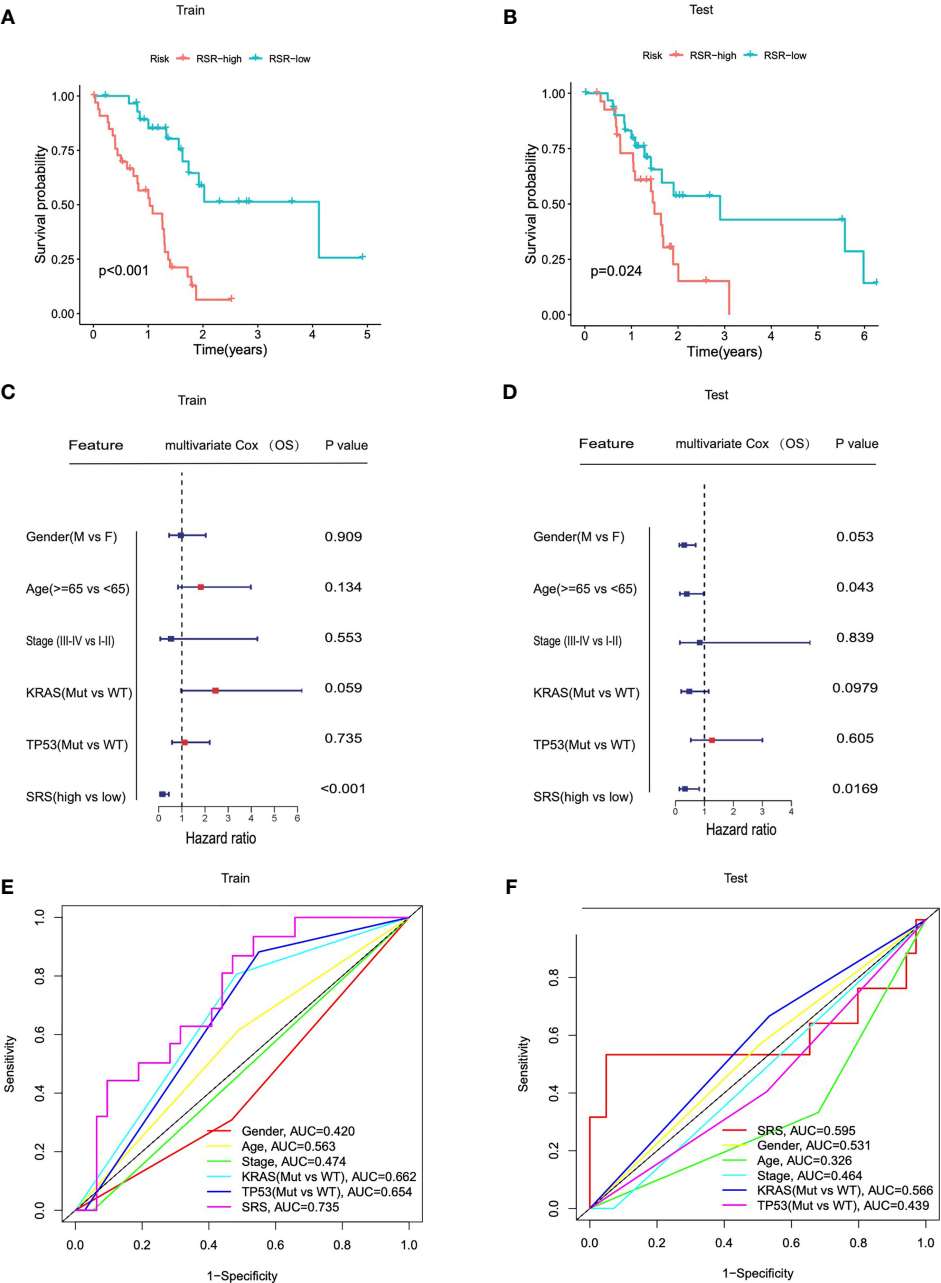
SRS served as a risk factor for overall survival in each cohort. **(A, B)** Kaplan–Meier analysis showed that patients of SRS-high exhibited worse OS in the TCGA training cohorts and testing cohorts. **(C, D)** Univariate Cox regression analysis and multivariate Cox regression analysis demonstrated that SRS was the only independent risk factor for OS among all the clinical variables in the TCGA training cohorts and testing cohorts. **(E, F)** Time-dependent ROC curves of the clinical variables at 3 years between patients at SRS-high and SRS-low in training and testing cohorts.

To evaluate the predictive abilities of our signature, we performed Kaplan–Meier survival and time-dependent ROC analysis in the training cohort (*n* = 62) and in the testing cohort (*n* = 62). In the training cohort, Kaplan–Meier analysis revealed that patients with higher SRS exhibited worse prognosis than those with lower scores (*p* < 0.0001, **Figure 4A**). Multivariate Cox regression analysis was performed on all clinical variables including gender (male or female), age (≥65 or <65), pathological stage (I–II or III–IV), KRAS status (mutation or wild type), TP53 status (mutation or wild type), and SRS (SRS-high or SRS-low), and results showed that SRS was the only independent risk factor among all the variables (*p* < 0.001, **Figure 4C**). The predictive accuracy of six clinical variables was 0.420, 0.563, 0.474, 0.662, 0.654, and 0.735 at 3 years (**Figure 4E**). In accordance with the results in the training cohort, a lower survival rate was observed in the SRS-high group in the testing cohort (*p* = 0.024, **Figure 4B**). In the testing cohort, among all clinical variables, multivariate Cox regression modeling indicated that age (*p* = 0.043) and SRS (*p* = 0.0169) were two independent risk factors for OS (**Figure 4D**). The predictive accuracy of six variables was 0.531, 0.326, 0.464, 0.566, 0.439, and 0.595 at 3 years (**Figure 4F**).

### Different Immune Characteristics and Mutational Patterns Were Observed Between SRS-High and -Low Cohorts

**Figure 5:**
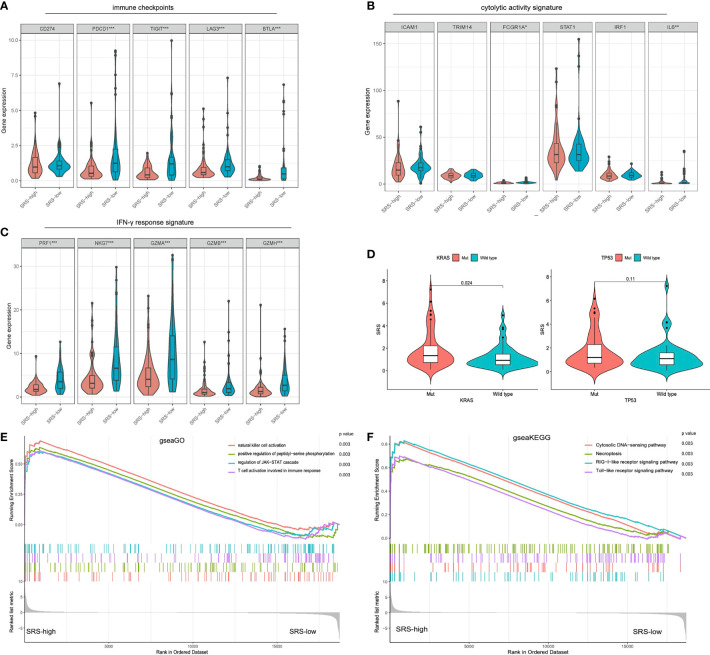
Different immune characteristics and mutational patterns were observed between SRS-high and SRS-low cohorts. **(A)** Compared with SRS-high samples, representative immune checkpoints including LAG3, TIGIT, BTLA, and PDCD1 were significantly elevated in IRS-low ones. **(B)** SRS-low cohort was characterized with significantly higher FGCR1 and IL-6 expression. **(C)** IFN-γ response signature of the SRS-low cohort was characterized with significantly higher PRF1, NKG7, GZMA, GZMB, and GZMH expression. **(D)** SRS was significantly elevated in KRAS^Mut^ or TP53^Mut^ samples compared with KRAS or TP53 wild-type samples. **(E, F)** gseaGo and gseaKEGG analysis demonstrated that SRS-low exhibited significantly higher activity of various immune processes compared with SRS-high. ssGSEA, single-sample gene set enrichment analysis; ***p < 0.001, **p < 0.01, *p < 0.05 and not significant (*p* > 0.05) by repeated measures with Wilcoxon test.

Considering that immune infiltration is always strongly associated with mutation in solid tumors, we investigated the immune characterization, mutational landscape, and their relationships in different PDACs. We performed further bioinformatic analyses to explore the genomic alterations, altered pathways, and immunogenicity alterations correlated with different immune-related risk groups. Compared with SRS-high samples, some representative immune checkpoints including PDCD1, TIGIT, LAG3, and BTLA were significantly elevated in SRS-low ones (**Figure 5A**). In addition, the IFN-γ response signature of the SRS-low cohort was characterized by significantly higher PRF1, NKG7, GZMA, GZMB, and GZMH expression (**Figure 5C**). Of note, the SRS-low group was closely associated with cytolytic activity signature, such as FGCR1 and IL-6, which was significantly elevated in the SRS-low cohort (**Figure 5B**), suggesting its plastic and therapeutically reprogrammable state. Then, as regards mutational features, SRS was significantly elevated in KRAS^Mut^ or TP53^Mut^ samples compared with KRAS and TP53 wild-type samples, the two significant mutated genes of PDAC patients (**Figure 5D**). To investigate the altered pathways underlying SRS in PDAC, DEGs between SRS-low and -high samples were identified and submitted for Gene Ontology enrichment analysis, respectively. gseaGo and gseaKEGG analysis demonstrated that SRS-low exhibited a markedly higher activity of various immune processes compared with SRS-high (**Figures 5E, F**). The top 4 significantly altered biological part (BP) embraced “natural killer cell activation”, “positive regulation of peptidyl-serine phosphorylation”, “T cell activation involved in immune response”, and “regulation of JAK-STAT cascade” among all the gene sets from Gene Ontology (**Figure 5E**). The most evidently altered KEGG pathway was presented as a “RIG-I-like receptor signaling pathway” among all the gene sets from KEGG (**Figure 5F**).

## Discussion

The primary tumor location of pancreatic cancer usually influences the outcome of clinical features, treatment, prognosis, and recurrence (3, 9–11). Pancreatic head cancer and pancreatic body/tail cancer are considered to be two different diseases and have altered outcomes. As for clinical presentation, most patients of pancreatic head cancer present jaundice early due to the obstruction of the common bile duct, while pancreatic body/tail cancer presents with weight loss and pain, symptoms more in keeping with advanced disease (36). Several studies have confirmed a different biological nature and survival (9, 11, 16). However, in recent years, the association between tumor location and prognosis has remained controversial for patients with different tumor resection. Previous studies suggested that patients with pancreatic head cancer have a better prognosis than those with pancreatic body/tail cancer, because patients with pancreatic head cancer have higher resection rates and greater utilization of adjuvant therapy, while those with pancreatic body/tail cancer have increased frequency of metastasis and lower resection rates due to late diagnosis (9, 17, 37). Nevertheless, some other studies have shown that patients with pancreatic head cancer have a poorer survival than those with pancreatic body/tail cancer, in that the tumor recurrence rate after curative resection is lower in pancreatic body/tail cancer (11, 17, 20). Yet, the exact mechanism has not been fully elucidated. Thus, the mechanism leading to the difference in prognosis between them needs to be further explored.

Firstly, our study based on 90 PDAC cases from our institution and 166 PDAC cases from the TCGA cohort indicated that the prognosis and the ORR for first-line treatment of patients with pancreatic body/tail cancer is superior to those with pancreatic head cancer. First-line treatment is a known prognostic factor for pancreatic cancer (38, 39). Moreover, our results demonstrated that the primary tumor site of PDAC was an independent risk factor for OS. Then, our research further confirmed with patients from the PDAC-TCGA cohort and tried to investigate the underlying biological activity of two subtypes. Consistently, our ssGSEA findings demonstrated that IL-6/JAK/STAT3 signaling was markedly enriched in pancreatic head tumors. We observed that accumulative evidence has demonstrated that the IL-6/JAK/STAT3 pathway is aberrantly hyperactivated in many types of cancer and such hyperactivation is generally associated with a poor clinical prognosis (40–42). In the TME, the IL-6/JAK/STAT3 pathway can have a profound effect on immune cell infiltration, exerting negative regulatory effects on neutrophils, natural killer (NK) cells, T cells, and dendritic cells (DCs), but positively regulating regulatory T (Treg) cells and myeloid-derived suppressor cell (MDSC) populations (43, 44). All in all, these effects contribute to a highly immunosuppressive TME.

Several studies have shown that the prognosis of tumor patients is closely related to the tumor immune microenvironment, and most of them have immune escape and immunosuppression, which is an essential factor in tumor progression (23, 45). Therefore, we further investigated the immune landscape in PDAC, and distinct immune characteristics were indeed observed between the two subtypes. Of note, we found increased infiltration of many types of immune cells, including immunosuppressive cells (Tregs, TAMs, and MDSCs) and anticancer immune response cells (CD8T cell and NK cell) in pancreatic head tumors. These lines of evidence indicated that the IL-6/JAK/STAT3 pathway is hyperactivated in pancreatic head tumors and leads to a dysregulated TME.

In recent years, despite the considerable amount of work done to develop gene signatures for its prognosis prediction (46, 47), quite a few studies have revealed the role of the anatomical site-related gene signature of certain molecular characteristics and prognostic factors for unfavorable survival in PDAC. Considering the unique molecular and clinical characteristics of pancreatic head tumors, it is essential to tailor specialized management for these patients. The aim of this study was to develop an efficient approach of prognosis prediction for pancreatic head tumors, and ultimately aid physicians in devising treatment strategies. Here, a univariate LASSO Cox regression model was used to screen robust prognostic biomarkers to establish an anatomical site-related gene signature, and the risk score derived from the anatomical site-related gene signature is called the SRS in our study; the five most important SRSs (CHL1A-S2, KRT8P30, AL365184.1, AC083841.2, and AC112255.1) were finally identified. Some biomarkers involved in our gene signature have been studied in many cancers. For instance, CHL1-AS2, one risk biomarker in our research, was negatively correlated to the ovarian cancer prognosis, which might be involved in the development of ovarian endometrium and high expression of lncRNA CHL1-AS2 upregulated in ovarian ectopic endometrium tissues (48, 49). In our study, CHL1-AS2 acts as a protective biomarker and serves as a novel therapeutic target for PDAC. Unfortunately, other biomarkers are rarely investigated in tumor progress and immune regulation, and whether the expression of these biomarkers affects the difference in immune characteristics of pancreatic head cancer needs further investigation. Furthermore, the combination of the anatomical site-related gene signature and clinicopathological features improves risk stratification and survival prediction, and the multivariate Cox regression analysis showed that SRS exhibited a considerable power of risk prediction for OS, even more significant than age, gender, stage, KRAS mutation, and TP53 mutation. As expected, multivariate Cox results jointly suggested that SRS developed specifically for pancreatic head tumor patients had a significantly better performance in prognostication compared with other clinicopathological features.

Subsequently, the prognostic value of this signature was validated in two independent cohorts: training (*n* = 62) and testing (*n* = 62) from TCGA. Our signature was proved to be the independent risk factor of PDAC prognosis, and time-dependent ROC curves of this signature and clinicopathological factors showed high AUC (0.735 and 0.595 in the training cohort and testing cohort, respectively) in predicting PDAC survival outcomes.

Immune infiltration abundance and the immune evasive mechanism are also characteristics of immune system dysregulation and play an important role in PDAC progression (50, 51). Aiming to interrupt the escape from immune surveillance, immunotherapeutic agents that target immune checkpoints including PD-L1/PD-1 and CTLA-4 have exhibited promising survival benefits in patients with metastatic melanoma, non-small cell lung cancer, and metastatic renal cancer in recent years (52–54). However, the potential relationships among immune infiltration, somatic mutations, and clinical outcomes in PDAC remain unclear. Therefore, in this study, the landscapes of immune checkpoints, cytolytic activity signature, IFN-γ signature, and somatic mutations were further investigated in the two types, and significantly different immune activities were observed between them, which indicated the differences in intrinsic tumor immunogenicity, while a higher mutation frequency of KRAS and TP53 was observed in the SRS-high group. Here, these results shed some light on the revelation of the immune and mutational landscape characterizations, and their relationships in PDAC of different anatomical sites. Thus, we further speculate that KRAS and TP53 mutations lead to IL-6/JAK/STAT3 pathway hyperactivation in pancreatic head cancer, and then affect the phenotype of the immune microenvironment in pancreatic head cancer, implying that this anatomical site-related signature is a promising and potent predictor of immunotherapy response in PDAC.

However, some limitations to our study should be acknowledged. First, this is a retrospective study; thus, the prognostic robustness and clinical usefulness of the anatomical site-related gene signature need further validation in larger prospective trials. Second, the TCGA cohort only contains few patients with higher tumor grades (G3 and G4) and pathologic stages (III and IV), which causes the failure in predicting PDAC prognosis in those subgroups. Thus, data from multicentric cohorts with comprehensive clinical information are needed to confirm our findings. Overall, we developed a robust gene signature that could predict the prognosis of PDAC. Moreover, a nomogram developed based on the signature with a strong capacity of predicting PDAC outcomes deserves promotion in clinical practice. The anatomical site-based model could be a useful tool to select SRS-low patients who may benefit from immunotherapy and SRS-high patients who may benefit from adjuvant therapies, and thus to facilitate personalized management of PDAC.

## Data Availability Statement

The raw data supporting the conclusions of this article will be made available by the authors, without undue reservation.

## Ethics Statement

The studies involving human participants were reviewed and approved by the Ethical Committee of Shanghai Jiao Tong University School of Medicine Affiliated Renji Hospital. The patients/participants provided their written informed consent to participate in this study.

## Author Contributions

WG and JM: Conceptualization, Methodology, Software, Formal analysis, Validation, Visualization, Investigation, and Writing—Original Draft. TM: Data Curation, Writing—Review and Editing. HX: Writing—Review and Editing, Project administration, and Funding acquisition. XFZ: Resources and Writing—Review and Editing. SL, YCW, JY, and MY: Data Curation. FJ, YW, MZ, TH, JH, and XZ: Resources. JC: Conceptualization, Writing—Review and Editing, Supervision, and Funding acquisition. LW: Conceptualization, Supervision, Project administration, and Funding acquisition. All authors contributed to the article and approved the submitted version.

## Funding

This work was supported by the National Key R&D Program of China (2019YFC1315900); the Clinical Research Plan of SHDC (No. SHDC2020CR1035B); the Innovation Group Project of Shanghai Municipal Health Commission (2019CXJQ03); the National Natural Science Foundation of China (81874048); Scientific and Technological Innovation Project of Science and Technology Commission of Shanghai Municipality (21JC1404300); Project from CSCO Clinical Oncology Research Foundation (Y-2019AZZD-0513); Shanghai Sailing Program (20YF1446400); the Shanghai Key Clinical Specialty (Oncology); the Shanghai Leading Talents Project; the Innovative Research Team of High-Level Local Universities in Shanghai; the Shanghai Municipal Commission of Health and Family Planning Grant 2018ZHYL0223; and Shanghai Municipal Education Commission-Gaofeng Clinical Medicine Grant Support (20161312).

## Conflict of Interest

The authors declare that the research was conducted in the absence of any commercial or financial relationships that could be construed as a potential conflict of interest.

## Publisher’s Note

All claims expressed in this article are solely those of the authors and do not necessarily represent those of their affiliated organizations, or those of the publisher, the editors and the reviewers. Any product that may be evaluated in this article, or claim that may be made by its manufacturer, is not guaranteed or endorsed by the publisher.

## Supplementary Material

Supplementary Table 1 Shows DEG between pancreatic head cancer and pancreatic body/tail cancer.

Supplementary Table 2 Shows the distribution of LASSO coefficients of the gene signature. DEGs, differentially expressed genes.

## References

[1] Sung H Ferlay J Siegel RL Laversanne M Soerjomataram I Jemal A. Global Cancer Statistics 2020: GLOBOCAN Estimates of Incidence and Mortality Worldwide for 36 Cancers in 185 Countries. CA Cancer J Clin (2021) 71(3):209–49. doi: 10.3322/caac.21660 33538338

[2] Siegel RL Miller KD Fuchs HE Jemal A. Cancer Statistics, 2021. CA Cancer J Clin (2021) 71(1):7–33. doi: 10.3322/caac.21654 33433946

[3] Mizrahi JD Surana R Valle JW Shroff RT. Pancreatic Cancer. Lancet (2020) 395(10242):2008–20. doi: 10.1016/S0140-6736(20)30974-0 32593337

[4] Fesinmeyer MD Austin MA Li CI De Roos AJ Bowen DJ. Differences in Survival by Histologic Type of Pancreatic Cancer. Cancer Epidemiol Biomarkers Prev (2005) 14(7):1766–73. doi: 10.1158/1055-9965.EPI-05-0120 16030115

[5] Ansari D Bauden M Bergström S Rylance R Marko-Varga G Andersson R. Relationship Between Tumour Size and Outcome in Pancreatic Ductal Adenocarcinoma. Br J Surg (2017) 104(5):600–7. doi: 10.1002/bjs.10471 28177521

[6] Yamamoto T Yagi S Kinoshita H Sakamoto Y Okada K Uryuhara K. Long-Term Survival After Resection of Pancreatic Cancer: A Single-Center Retrospective Analysis. World J Gastroenterol (2015) 21(1):262–8. doi: 10.3748/wjg.v21.i1.262 PMC4284344 25574100

[7] Luo G Jin K Deng S Cheng H Fan Z Gong Y. Roles of CA19-9 in Pancreatic Cancer: Biomarker, Predictor and Promoter. Biochim Biophys Acta Rev Cancer (2021) 1875(2):188409. doi: 10.1016/j.bbcan.2020.188409 32827580

[8] Dreyer SB Chang DK Bailey P Biankin AV. Pancreatic Cancer Genomes: Implications for Clinical Management and Therapeutic Development. Clin Cancer Res (2017) 23(7):1638–46. doi: 10.1158/1078-0432.CCR-16-2411 28373362

[9] Artinyan A Soriano PA Prendergast C Low T Ellenhorn JD Kim J. The Anatomic Location of Pancreatic Cancer Is a Prognostic Factor for Survival. HPB (Oxford). (2008) 10(5):371–6. doi: 10.1080/13651820802291233 PMC2575681 18982154

[10] Matsuno S Egawa S Fukuyama S Motoi F Sunamura M Isaji S. Pancreatic Cancer Registry in Japan: 20 Years of Experience. Pancreas (2004) 28(3):219–30. doi: 10.1097/00006676-200404000-00002 15084961

[11] Lau MK Davila JA Shaib YH. Incidence and Survival of Pancreatic Head and Body and Tail Cancers: A Population-Based Study in the United States. Pancreas (2010) 39(4):458–62. doi: 10.1097/MPA.0b013e3181bd6489 19924019

[12] Nakata B Yamada N Amano R Tendo M Inoue M Sakurai K. Comparison of Clinicopathological Characteristics of Curatively Resected Pancreatic Head and Body/Tail Ductal Cancers. J Exp Clin Cancer Res (2007) 26(4):459–66. 18365539

[13] Schmidt-Hansen M Berendse S Hamilton W. Symptoms of Pancreatic Cancer in Primary Care: A Systematic Review. Pancreas (2016) 45(6):814–8. doi: 10.1097/MPA.0000000000000527 26495795

[14] Palanivelu C Jani K Senthilnathan P Parthasarathi R Rajapandian S Madhankumar MV. Laparoscopic Pancreaticoduodenectomy: Technique and Outcomes. J Am Coll Surg (2007) 205(2):222–30. doi: 10.1016/j.jamcollsurg.2007.04.004 17660068

[15] McGuigan A Kelly P Turkington RC Jones C Coleman HG McCain RS. Pancreatic Cancer: A Review of Clinical Diagnosis, Epidemiology, Treatment and Outcomes. World J Gastroenterol (2018) 24(43):4846–61. doi: 10.3748/wjg.v24.i43.4846 PMC6250924 30487695

[16] Watanabe I Sasaki S Konishi M Nakagohri T Inoue K Oda T. Onset Symptoms and Tumor Locations as Prognostic Factors of Pancreatic Cancer. Pancreas (2004) 28(2):160–5. doi: 10.1097/00006676-200403000-00007 15028948

[17] Winer LK Dhar VK Wima K Morris MC Lee TC Shah SA. The Impact of Tumor Location on Resection and Survival for Pancreatic Ductal Adenocarcinoma. J Surg Res (2019) 239:60–6. doi: 10.1016/j.jss.2019.01.061 30802706

[18] Zheng Z Wang M Tan C Chen Y Ping J Wang R. Disparities in Survival by Stage After Surgery Between Pancreatic Head and Body/Tail in Patients With Nonmetastatic Pancreatic Cancer. PloS One (2019) 14(12):e0226726. doi: 10.1371/journal.pone.0226726 31856205 PMC6922472

[19] Cancer Genome Atlas Research Network. Integrated Genomic Characterization of Pancreatic Ductal Adenocarcinoma. Cancer Cell (2017) 32(2):185–203.e13. doi: 10.1016/j.ccell.2017.07.007 28810144 PMC5964983

[20] Yin L Xiao L Gao Y Wang G Gao H Peng Y. Comparative Bioinformatical Analysis of Pancreatic Head Cancer and Pancreatic Body/Tail Cancer. Med Oncol (2020) 37(5):46. doi: 10.1007/s12032-020-01370-0 32277286

[21] O'Reilly EM Oh DY Dhani N Renouf DJ Lee MA Sun W. Durvalumab With or Without Tremelimumab for Patients With Metastatic Pancreatic Ductal Adenocarcinoma: A Phase 2 Randomized Clinical Trial. JAMA Oncol (2019) 5(10):1431–8. doi: 10.1001/jamaoncol.2019.1588 PMC6647002 31318392

[22] Upadhrasta S Zheng L. Strategies in Developing Immunotherapy for Pancreatic Cancer: Recognizing and Correcting Multiple Immune "Defects" in the Tumor Microenvironment. J Clin Med (2019) 8(9):1472. doi: 10.3390/jcm8091472 PMC6780937 31527414

[23] Lei X Lei Y Li JK Du WX Li RG Yang J. Immune Cells Within the Tumor Microenvironment: Biological Functions and Roles in Cancer Immunotherapy. Cancer Lett (2020) 470:126–33. doi: 10.1016/j.canlet.2019.11.009 31730903

[24] Liberzon A Birger C Thorvaldsdóttir H Ghandi M Mesirov JP Tamayo P. The Molecular Signatures Database (MSigDB) Hallmark Gene Set Collection. Cell Syst (2015) 1(6):417–25. doi: 10.1016/j.cels.2015.12.004 PMC4707969 26771021

[25] Barbie DA Tamayo P Boehm JS Kim SY Moody SE Dunn IF. Systematic RNA Interference Reveals That Oncogenic KRAS-Driven Cancers Require TBK1. Nature (2009) 462(7269):108–12. doi: 10.1038/nature08460 PMC2783335 19847166

[26] Jie Y Gong J Xiao C Zheng J Zhang Z Li X. The Clinical Value of Fibulin-1 for Prognosis and Its Prospective Mechanism in Intrahepatic Cholangiocarcinoma. HPB (Oxford). (2019) 21(4):499–507. doi: 10.1016/j.hpb.2018.09.002 30266493

[27] Xiao C Gong J Jie Y Cao J Chen Z Li R. NCAPG Is a Promising Therapeutic Target Across Different Tumor Types. Front Pharmacol (2020) 11:387. doi: 10.3389/fphar.2020.00387 32300299 PMC7142249

[28] Yoshihara K Shahmoradgoli M Martínez E Vegesna R Kim H Torres-Garcia W. Inferring Tumour Purity and Stromal and Immune Cell Admixture From Expression Data. Nat Commun (2013) 4:2612. doi: 10.1038/ncomms3612 24113773 PMC3826632

[29] Chen B Khodadoust MS Liu CL Newman AM Alizadeh AA. Profiling Tumor Infiltrating Immune Cells With CIBERSORT. Methods Mol Biol (2018) 1711:243–59. doi: 10.1007/978-1-4939-7493-1_12 PMC5895181 29344893

[30] Langfelder P Horvath S. WGCNA: An R Package for Weighted Correlation Network Analysis. BMC Bioinf (2008) 9:559. doi: 10.1186/1471-2105-9-559 PMC2631488 19114008

[31] Ritchie ME Phipson B Wu D Hu Y Law CW Shi W. Limma Powers Differential Expression Analyses for RNA-Sequencing and Microarray Studies. Nucleic Acids Res (2015) 43(7):e47. doi: 10.1093/nar/gkv007 25605792 PMC4402510

[32] Tibshirani R. The Lasso Method for Variable Selection in the Cox Model. Stat Med (1997) 16(4):385–95. doi: 10.1002/(SICI)1097-0258(19970228)16:4<385::AID-SIM380>3.0.CO;2-3 9044528

[33] Heagerty PJ Lumley T Pepe MS. Time-Dependent ROC Curves for Censored Survival Data and a Diagnostic Marker. Biometrics (2000) 56(2):337–44. doi: 10.1111/j.0006-341X.2000.00337.x 10877287

[34] Subramanian A Tamayo P Mootha VK Mukherjee S Ebert BL Gillette MA. Gene Set Enrichment Analysis: A Knowledge-Based Approach for Interpreting Genome-Wide Expression Profiles. Proc Natl Acad Sci USA (2005) 102(43):15545–50. doi: 10.1073/pnas.0506580102 PMC1239896 16199517

[35] Yu G Wang LG Han Y He QY. Clusterprofiler: An R Package for Comparing Biological Themes Among Gene Clusters. Omics (2012) 16(5):284–7. doi: 10.1089/omi.2011.0118 PMC3339379 22455463

[36] Walter FM Mills K Mendonça SC Abel GA Basu B Carroll N. Symptoms and Patient Factors Associated With Diagnostic Intervals for Pancreatic Cancer (SYMPTOM Pancreatic Study): A Prospective Cohort Study. Lancet Gastroenterol Hepatol (2016) 1(4):298–306. doi: 10.1016/S2468-1253(16)30079-6 28404200 PMC6358142

[37] Bilici A. Prognostic Factors Related With Survival in Patients With Pancreatic Adenocarcinoma. World J Gastroenterol (2014) 20(31):10802–12. doi: 10.3748/wjg.v20.i31.10802 PMC4138460 25152583

[38] Glatzer M Panje CM Sirén C Cihoric N Putora PM. Decision Making Criteria in Oncology. Oncology (2020) 98(6):370–8. doi: 10.1159/000492272 30227426

[39] Panje CM Glatzer M von Rappard J Rothermundt C Hundsberger T Zumstein V. Applied Swarm-Based Medicine: Collecting Decision Trees for Patterns of Algorithms Analysis. BMC Med Res Methodol (2017) 17(1):123. doi: 10.1186/s12874-017-0400-y 28814269 PMC5559810

[40] Kumari N Dwarakanath BS Das A Bhatt AN. Role of Interleukin-6 in Cancer Progression and Therapeutic Resistance. Tumour Biol (2016) 37(9):11553–72. doi: 10.1007/s13277-016-5098-7 27260630

[41] Chen Y Wang J Wang X Liu X Li H Lv Q. STAT3, a Poor Survival Predicator, Is Associated With Lymph Node Metastasis From Breast Cancer. J Breast Cancer. (2013) 16(1):40–9. doi: 10.4048/jbc.2013.16.1.40 PMC3625768 23593080

[42] Johnson DE O'Keefe RA Grandis JR. Targeting the IL-6/JAK/STAT3 Signalling Axis in Cancer. Nat Rev Clin Oncol (2018) 15(4):234–48. doi: 10.1038/nrclinonc.2018.8 PMC5858971 29405201

[43] Yu H Pardoll D Jove R. STATs in Cancer Inflammation and Immunity: A Leading Role for STAT3. Nat Rev Cancer. (2009) 9(11):798–809. doi: 10.1038/nrc2734 19851315 PMC4856025

[44] Kujawski M Zhang C Herrmann A Reckamp K Scuto A Jensen M. Targeting STAT3 in Adoptively Transferred T Cells Promotes Their *In Vivo* Expansion and Antitumor Effects. Cancer Res (2010) 70(23):9599–610. doi: 10.1158/0008-5472.CAN-10-1293 PMC3017475 21118964

[45] Nakamura K Smyth MJ. Myeloid Immunosuppression and Immune Checkpoints in the Tumor Microenvironment. Cell Mol Immunol (2020) 17(1):1–12. doi: 10.1038/s41423-019-0306-1 31611651 PMC6952382

[46] Huang X Zhang G Tang T Liang T. Identification of Tumor Antigens and Immune Subtypes of Pancreatic Adenocarcinoma for mRNA Vaccine Development. Mol Cancer. (2021) 20(1):44. doi: 10.1186/s12943-021-01310-0 33648511 PMC7917175

[47] Moffitt RA Marayati R Flate EL Volmar KE Loeza SG Hoadley KA. Virtual Microdissection Identifies Distinct Tumor- and Stroma-Specific Subtypes of Pancreatic Ductal Adenocarcinoma. Nat Genet (2015) 47(10):1168–78. doi: 10.1038/ng.3398 PMC4912058 26343385

[48] Zhang C Wu W Ye X Ma R Luo J Zhu H. Aberrant Expression of CHL1 Gene and Long non-Coding RNA CHL1-AS1, CHL1-AS2 in Ovarian Endometriosis. Eur J Obstet Gynecol Reprod Biol (2019) 236:177–82. doi: 10.1016/j.ejogrb.2019.03.020 30943448

[49] Geisler S Coller J. RNA in Unexpected Places: Long Non-Coding RNA Functions in Diverse Cellular Contexts. Nat Rev Mol Cell Biol (2013) 14(11):699–712. doi: 10.1038/nrm3679 24105322 PMC4852478

[50] Al-Abdallah A Jahanbani I Mehdawi H Ali RH Al-Brahim N Mojiminiyi O. Down-Regulation of the Human Major Histocompatibility Complex Class I Chain-Related Gene A (MICA) and Its Receptor Is Mediated by microRNA-146b-5p and Is a Potential Mechanism of Immunoediting in Papillary Thyroid Carcinoma. Exp Mol Pathol (2020) 113:104379. doi: 10.1016/j.yexmp.2020.104379 31935378

[51] Stenman A Backman S Johansson K Paulsson JO Stålberg P Zedenius J. Pan-Genomic Characterization of High-Risk Pediatric Papillary Thyroid Carcinoma. Endocr Relat Cancer (2021) 28(5):337–51. doi: 10.1530/ERC-20-0464 PMC8111328 33827048

[52] Hodi FS O'Day SJ McDermott DF Weber RW Sosman JA Haanen JB. Improved Survival With Ipilimumab in Patients With Metastatic Melanoma. N Engl J Med (2010) 363(8):711–23. doi: 10.1056/NEJMoa1003466 PMC3549297 20525992

[53] Topalian SL Hodi FS Brahmer JR Gettinger SN Smith DC McDermott DF. Safety, Activity, and Immune Correlates of Anti-PD-1 Antibody in Cancer. N Engl J Med (2012) 366(26):2443–54. doi: 10.1056/NEJMoa1200690 PMC3544539 22658127

[54] Choueiri TK Escudier B Powles T Mainwaring PN Rini BI Donskov F. Cabozantinib Versus Everolimus in Advanced Renal-Cell Carcinoma. N Engl J Med (2015) 373(19):1814–23. doi: 10.1056/NEJMoa1510016 PMC5024539 26406150

